# Genetic and clinical characteristics including occurrence of testicular adrenal rest tumors in Slovak and Slovenian patients with congenital adrenal hyperplasia due to 21-hydroxylase deficiency

**DOI:** 10.3389/fendo.2023.1134133

**Published:** 2023-03-17

**Authors:** Robert Saho, Vita Dolzan, Mojca Zerjav Tansek, Andrea Pastorakova, Robert Petrovic, Maria Knapkova, Katarina Trebusak Podkrajsek, Jasna Suput Omladic, Sara Bertok, Magdalena Avbelj Stefanija, Primoz Kotnik, Tadej Battelino, Zuzana Pribilincova, Urh Groselj

**Affiliations:** ^1^ Department of Pediatrics and Inherited Metabolic Disorders, First Faculty of Medicine, Charles University and General University Hospital in Prague, Prague, Czechia; ^2^ Faculty of Medicine, Comenius University, Bratislava, Slovakia; ^3^ Faculty of Medicine, Institute of Biochemistry and Molecular Genetics, University of Ljubljana, Ljubljana, Slovenia; ^4^ Faculty of Medicine, University of Ljubljana, Ljubljana, Slovenia; ^5^ Department of Endocrinology, Diabetes, and Metabolic Diseases, University Children’s Hospital, University Medical Centre Ljubljana, Ljubljana, Slovenia; ^6^ Genetics and Clinical Genetics, Faculty of Medicine, Institute of Medical Biology, Comenius University, Bratislava, Slovakia; ^7^ Neonatal Screening Centre (NSC) of SR Banská Bystrica, Children University Hospital (CHUH), Banská Bystrica, Slovakia; ^8^ Clinical Institute for Special Laboratory Diagnostics, University Children’s Hospital, University Medical Centre Ljubljana, Ljubljana, Slovenia; ^9^ Department of Pediatrics, Faculty of Medicine, National Institute of Children’s Diseases, Comenius University in Bratislava, Bratislava, Slovakia

**Keywords:** congenital adrenal hyperplasia, CAH, CYP21A2, genotype-phenotype, 21 hydroxylase deficiency, 21-OH deficiency, newborn screening, testicular adrenal rest tumors (TART)

## Abstract

**Objective:**

To analyze the mutational spectrum, clinical characteristics, genotype–phenotype correlations, testicular adrenal rests tumor prevalence, and role of neonatal screening in congenital adrenal hyperplasia (CAH) patients from Slovakia and Slovenia.

**Design and methods:**

Data were obtained from 104 patients with CAH registered in Slovak and Slovenian databases. Low-resolution genotyping was performed to detect the most common point mutations. To detect deletions, conversions, point mutations, or other sequence changes in the *CYP21A2* gene, high-resolution genotyping was performed. Genotypes were classified according to residual 21-hydroxylase activity (null, A, B, C).

**Results:**

64% of the individuals had the salt-wasting form (SW-CAH), 15% the simple virilizing form (SV-CAH), and 21% the non-classic (NC-CAH). *CYP21A2* gene deletion/conversion and c.293-13A/C>G pathogenic variant accounted together for 55.5% of the affected alleles. In SV-CAH p.Ile172Asn was the most common pathogenic variant (28.13%), while in NC-CAH p.Val282Leu (33.33%), *CYP21A2* gene deletion/conversion (21.43%), c.293-13A/C>G (14.29%), Pro30Leu (11.90%). The frequency of alleles with multiple pathogenic variants was higher in Slovenian patients (15.83% of all alleles). Severe genotypes (0 and A) correlated well with the expected phenotype (SW in 94.74% and 97.3%), while less severe genotypes (B and C) correlated weaklier (SV in 50% and NC in 70.8%). The median age of SW-CAH patients at the time of diagnosis was 6 days in Slovakia vs. 28.5 days in Slovenia (p=0.01). Most of the Slovak patients in the cohort were detected by NBS. (24 out of 29). TARTs were identified in 7 out of 24 male patients, of whom all (100%) had SW-CAH and all had poor hormonal control. The median age at the diagnosis of TARTs was 13 years.

**Conclusion:**

The study confirmed the importance of neonatal screening, especially in the speed of diagnosis of severe forms of CAH. The prediction of the 21-OH deficiency phenotype was reasonably good in the case of severe pathogenic variants, but less reliable in the case of milder pathogenic variants, which is consistent compared to data from other populations. Screening for TARTs should be realized in all male patients with CAH, since there is possible remission when identified early.

## 1 Introduction

Congenital adrenal hyperplasia (CAH; incidence 1:14000–18000) is an autosomal recessive disorder, mostly caused by a 21-hydroxylase deficiency (21-OH) (1, 2). A complete loss of 21-OH function results in the most severe salt-wasting (SW-CAH) phenotypes, whereas a minimal residual 21-OH production is sufficient to maintain aldosterone homeostasis, resulting in moderate simple virilizing phenotypes (SV-CAH) or mild less-symptomatic nonclassical CAH phenotypes (NC-CAH) (1, 2). Numerous studies have examined large nation-based or population-based cohorts of patients around the world to establish genotype-phenotype associations (3–9). *CYP21A2* is located near its pseudogene (*CYP21A2P*), which is 96% homologous to it. Up to 75% of CAH pathogenic variants result from gene recombination and gene conversion events in this region. The majority of pathogenic variants are inherited, while only a small fraction are *de novo* mutations (10).

It is important to study *CYP21A2* molecular genetics and the genotype-phenotype correlation of pathogenic variants to develop relevant and effective newborn screening (NBS) programs that may prevent neonatal salt crisis consequently reducing morbidity and mortality (2, 11). Nationwide NBS for CAH has been performed in Slovakia since 2003. In 2020, 56,756 neonates were screened and 4 cases of CAH were detected. Since 2003 951,200 newborns have been screened and 80 cases of CAH have been detected in Slovakia. (incidence 1:11 890). In Slovenia NBS for CAH is planned to be introduced in 2023 (12).

Testicular adrenal rest tumors (TARTs) are a common complication typically in classic CAH male patients with a prevalence from 14% to 86%, with an average of 25% in adolescents, and 46% in men, they also occur in children, with an increase in prevalence during puberty (1, 13). TART tissue has adrenal as well as testicular characteristics and can produce steroids. Poor hormonal control with elevated ACTH, 17-hydroxyprogesterone (17-OHP), and androstenedione concentrations seems to be associated with TARTs. However, TARTs also occur in well-controlled patients and only a few studies have found a clear association between hormonal control and TARTs. TARTs can cause severe pain complaints and irreversible damage to the testicular parenchyma, leading to infertility (13).

The study aimed to systematically analyze the mutational spectrum, clinical characteristics, genotype–phenotype correlations, and TARTs prevalence in 104 CAH patients from Slovakia and Slovenia. Furthermore, we compared the clinical characteristics of screened and unscreened patients, as one country performs NBS for CAH, while the other does not.

## 2 Materials and methods

**Figure 1:**
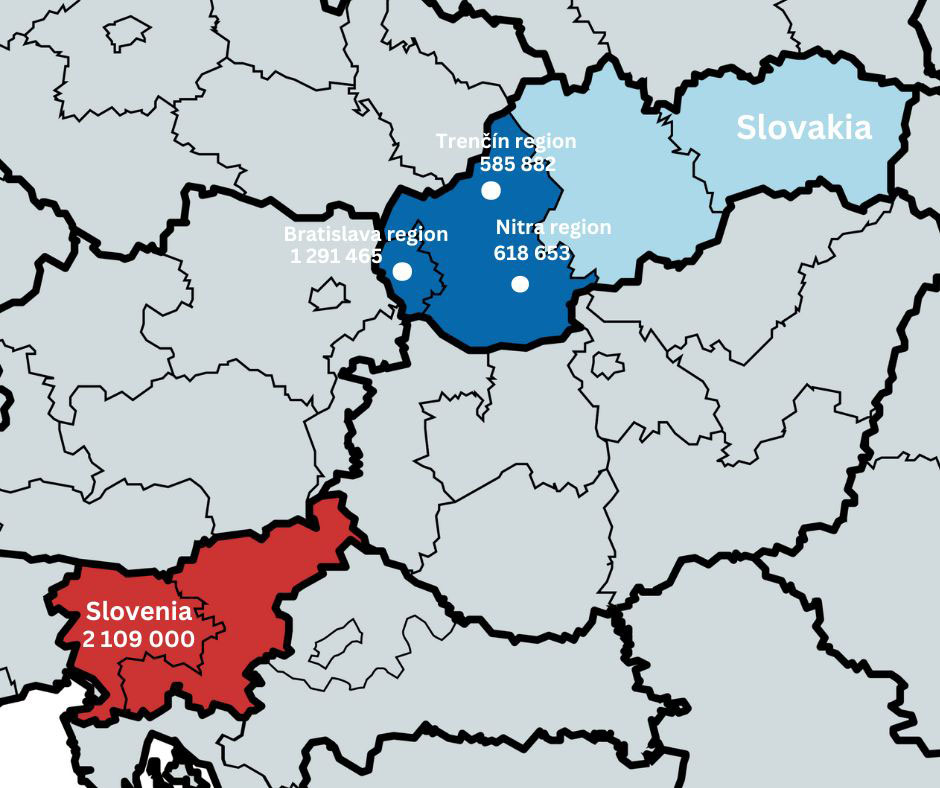
The map showing the origin of Slovak and Slovenian cohorts.

The studied population included individuals followed up with a diagnosis of CAH at the Department of Paediatrics, Comenius University, Bratislava, Slovakia (n=40) and the Department of Pediatric Endocrinology, Diabetes and Metabolic Diseases (DPEDMD), University Children’s Hospital, Ljubljana, Slovenia (n=60). Patients from the Slovak cohort originated from the capital, Bratislava, and the western region of Slovakia which has a total population of 2,496,000 people (**Figure 1**). The DPEDMD serves as the nationwide reference center for CAH in Slovenia which has a total population of 2,109,000 (**Figure 1**). Individuals included in the cohort were followed from 1984 in Slovenia and from 1996 in Slovakia until 2021 when the cohort data were evaluated. Clinical and laboratory data on the patients were obtained by retrospective data collection from the medical records in both countries. The study protocol was approved by the National Medical Ethics Committee of Slovenia (No.: 0120-290/2021/3).

### 2.1 Clinical classification/phenotyping

In all patients, the diagnosis of CAH was made or verified by a pediatric endocrinologist, based on the history, physical examination, electrolyte, and hormonal data. Serum 17-OHP values were available for Slovenian and Slovak patients before the introduction of NBS. Slovak patients born after the introduction of NBS for CAH had SW-CAH assigned based on high concentrations of 17-OHP measured from a dry drop of capillary blood on filter paper using an immunofluorescence method, supplemented with a mineralogram, serum steroid values, and in some patients renin.

In NBS for CAH, the screening marker is 17-OHP, which can detect 21-hydroxylase deficiency early and reliably. 17-OHP as a precursor of stress hormones is often elevated in newborns with other perinatal complications than CAH. Its concentration also depends on the maturity and birth weight of the newborn. Because of the potential for false positivity, a method was introduced in a pilot study to divide neonates according to maturity and weight into 4 categories with respect to the cut-off limit for 17-OHP. The reference values determined in this way will allow capturing all positive cases of 21-hydroxylase deficiency with a low percentage of false positive cases. In addition to correctly defined cut-off limits, the category of rescreenings - repeated screenings - is important. The selected group of newborns has been evaluated for NBS only after two samples. The first collection of a dry blood sample on the 4th day of life, the second collection on the 10th-14th day of life (12).

The criteria for SW-CAH in addition to basal 17-OHP levels were: either salt crisis in the neonatal period or/and elevated plasma renin activity (PRA), hyponatremia and hyperkalemia, virilized genitalia in girls. Treatment for the SW-CAH consisted of hydrocortisone (HC), fludrocortisone (FC), and salt replacement. For SV-CAH the criteria were: absence of salt wasting disorder, genital virilization in girls, gonadotropin-independent precocious puberty, accelerated growth and advanced bone age, and high 17-OHP levels on repeated sampling or after ACTH stimulation. Treatment was mostly limited to hydrocortisone replacement and low doses of mineralocorticoids. In girls, NC-CAH was diagnosed when they had no or minimal genital virilization, further had precocious pubarche, hirsutism or other signs of hyperandrogenemia and high 17-OHP levels after ACTH stimulation. In boys, NC-CAH was diagnosed if precocious puberty/virilization occurred after 4th year of life along with high levels of 17-OHP after ACTH stimulation (3, 7, 14).

In boys diagnosed with CAH, TARTs were diagnosed by ultrasonographic (USG) evaluation performed by paediatric radiologists with experience in scrotal ultrasound. Male patients were approximately once every 2 years subjected to scrotal ultrasound for TARTs screening. Longitudinal and transverse axes of the testes and TARTs, when visible, were measured.

### 2.2 *CYP21A2* gene analysis

A two-step genotyping approach was used for *CYP21A2* gene analysis. Low-resolution genotyping (qPCR, MLPA, and SnaPshot analysis) was performed to detect the most common pathogenic variants. High-resolution genotyping including Southern blotting, allele-specific long-range PCR, SNPCheck, and sequencing was performed to detect gene deletions, conversions, point mutations, or other sequence changes in the *CYP21A2* gene. The genotyping approach is described in detail elsewhere (7, 12, 15). CAH genotype-phenotype correlations were predicted based on Speiser´s classification of pathogenic variants into four mutation groups: null (SW-CAH), A (SW-CAH/SV-CAH), B (SV-CAH), C (NC-CAH) (16). In addition, the genotype of a novel pathogenic variant with no information about impairment of 21-OH function on at least one allele was assigned as D and excluded from the genotype-phenotype correlation analysis. As CAH is caused by homozygous or compound heterozygous pathogenic variants, the allele coding for the protein with greater residual 21-OH activity usually determines the severity of the overall genotype. Therefore, the haplotypes containing multiple pathogenic variants were grouped based on the most severe pathogenic variant.

### 2.3 Statistical analysis

Continuous data with normal distribution are presented as mean or median value and standard deviation (SD). For categorical data, percentages are reported. T-tests were used to compare means. Categorical variables were compared by Fisher’s exact test. A *P*-value below 0.05 was considered statistically significant.

## 3 Results

Our database included 104 patients. As genotype and phenotype information was complete for 100 patients (96.15%), all the analyses were performed in a cohort consisting of 100 patients. The genotype-phenotype correlations were analyzed in 98 patients, as we excluded 2 patients with group D genotypes. The main characteristics of the cohort are summarized in **Table 1**.

**Table 1:** The main characteristics of the cohort (n = 100).

	Slovakia (Western region)	Slovenia	p-value
**Probands in total (n)**	40 (25 girls, 15 boys)	60 (32 girls, 28 boys)	0.414
**Mean current age (years)**	11.5	19.5	0.000001
**SW-CAH in total (n)**	29 (18 girls, 11 boys)	34 (14 girls, 20 boys)	0.134
SW-CAH - median age at dg.	6 days (3 to 30 days)	28.5 days (0 to 120 days)	0.011
SW-CAH diagnosed by screening (n)	24 (14 girls, 10 boys)	NA	NA
SW-CAH screened - median age at dg.	5 days	NA	NA
SW-CAH average 17-OHP at dg. + SD (ng/ml)	563.9 ± 1001.2(dry blood)	671.7± 1041.5 (venous blood)	0.356
**SV-CAH in total**	5 (1 girl, 4 boys)	11 (6 girls, 5 boys)	0.58
SV-CAH - median age at dg.	0.74 years (4 days -10.5 years)	1.65 years (2.79 – 7 years)	0.498
SV-CAH diagnosed by screening	2 (1 girl, 1 boy)	NA	NA
SV-CAH screened - median age at dg.	6.5 days	NA	NA
SV-CAH average 17-OHP at dg. + SD (ng/ml)	51.8 ± 14 (dry blood)	1388.8 ± 1959.7 (venous blood)	0.057
**NC-CAH**	6 (6 girls)	15 (12 girls, 3 boys)	0.318
NC-CAH - median age at dg.	7.19 years	7.2 years	0.445
NC-CAH average 17-OHP at dg. + SD (ng/ml)	38 ± 22.7(venous blood)	116 ± 117.8 (venous blood)	0.012
**TARTS in male patients** (%)*	33.3% (2 out of 6)	27.8% (5 out of 18)	1
SW-CAH in patients with TARTs*	2 (100%)	5 (100%)	1
Median age at dg. of CAH in patients with TARTs*	16.5 days	15 days	0,44
Median age at dg. of TARTs*	13 years		
Avare 17-OHP at dg. of TARTs*	217.88 nmol/l		

* information on scrotal ultrasound available from 24 male probands.

CAH, congenital adrenal hyperplasia; SW-CAH, salt-wasting form; SV-CAH, simple virilizing form; NC-CAH, non-classic form; dg, diagnosis; 17-OHP, 17-hydroxyprogesterone; SD, standard deviation; TART, testicular adrenal rest tumour; NA, not applicable.

In our cohort, there were a total of 57 female patients, of which 32 (56.14%) had SW-CAH, 18 (31.58%) NC-CAH, and 7 (12.28%) SV-CAH. There were a total of 43 male patients in the cohort, of which 31 (72.09%) had SW-CAH, 9 SV-CAH, and 3 (6.98%) NC-CAH.

The median age of SW-CAH patients at the time of diagnosis was 6 days in Slovakia vs. 28.5 days in Slovenia (p=0.01). Most of the Slovak patients in the cohort were detected by NBS. (24 out of 29). The median age at diagnosis of Slovak SV-CAH patients was 0.74 years, and in Slovenian 1.65 years.

Complete genotypes and clinical phenotypes of the cohort are shown in **Supplementary Table 1**. Among 100 patients 63% presented with SW-CAH (31 boys and 32 girls), 16% had SV-CAH (9 boys and 7 girls) and 21% had NC-CAH (3 boys and 18 girls). Most patients in our cohort were hemizygous (35%), with a complete deletion of the *CYP21A2* gene or a large deletion/conversion on one allele, 34% were compound heterozygotes and 31% were homozygous. The most common pathogenic variant in SW-CAH were c.293-13A/C>G (44.53%) and *CYP21A2* gene deletion/conversion (32.03%). In SV-CAH, the most common pathogenic variant was p.Ile172Asn (28.13%), but it was also observed in 3.91% of SW-CAH alleles as well as in 7.14% of NC-CAH patients’ alleles. Other common pathogenic variants found in SV-CAH were c.293-13A/C>G in 21.88% of alleles, *CYP21A2* gene deletion/conversion in 15.63% of alleles, and Pro30Leu in 12.5% of alleles of SV-CAH patients. Together with p.Ile172Asn, these pathogenic variants accounted for 78.13% of SV-CAH. The most common pathogenic variants in NC-CAH patients were p.Val282Leu (33.33%), *CYP21A2* gene deletion/conversion (21.43%), c.293-13A/C>G (14.29%) Pro30Leu (11.90%), which together accounted for more 80% of NC-CAH. p.Val282Leu pathogenic variant also represented the least severe pathogenic variant in 6.25% of SV-CAH patients.

As summarized in **Supplementary Table 2**, a good correlation between genotype and phenotype was observed in patients with severe pathogenic variants assigned to mutation groups 0 and A (94.74% for mutation group 0 and 97.92% for mutation group A). Greater variability in clinical phenotypes was observed in patients with less severe pathogenic variants, particularly in carriers of p.Ile172Asn and Pro30Leu, assigned to mutation groups B and C, respectively. (62.5% of relative frequency of the predicted phenotype for mutation group B and 75.28% for mutation group C). In most patients, the observed phenotype corresponded to the severity of the less severely affected of the two alleles; however, the presence of a group 0 or A pathogenic variants on the other allele often led to more severe clinical manifestations.

*CYP21A2* allele frequencies in our cohort are shown in **Table 2**. Only 2 pathogenic variants: c.293-13A/C>G and *CYP21A2* gene deletion/conversion occurred with a frequency greater than 20%. Together they accounted for more than half (55.5%) of the affected alleles in the cohort. The other 2 common pathogenic variants occurred at a frequency of 8%, namely p.Ile172Asn and p.Val282Leu. Country-specific differences were observed in the frequencies of occurrence of a particular pathogenic variant. The frequency of *CYP21A2* gene deletion was 24.5% in the whole cohort, 25.8% in Slovenian patients, and 22.5% in Slovak patients. The c.293-13A/C>G pathogenic variant with an allele frequency of 31% in the whole cohort was the most frequent pathogenic variant in both Slovak (37.5%) and Slovenian patients (26.7%).

**Table 2:** *CYP21A2* allele frequencies in individual populations of the cohort.

*CYP21A2* allele	Slovakia	Slovenia	Together	
	n	n	n	%
del/conv.	18	31	49	24.5%
prom.conv.	1	1	2	1.0%
c.293-13C/A>G	30	32	62	31.0%
p.Pro31Leu	4	5	9	4.5%
del 8bp (ex3)	1	2	3	1.5%
p.Ile172Asn	6	10	16	8.0%
p.Val282Leu	5	11	16	8.0%
cluster ex6	0	1	1	0.5%
p.Arg409Cys	1	0	1	0.5%
p.Arg317Ter	1	0	1	0.5%
p.Arg357Trp	1	1	2	1.0%
p.Arg355Cys	1	0	1	0.5%
p.Arg357GIn	2	0	2	1.0%
p.Arg484Pro	0	1	1	0.5%
p.Gln319Ter	2	2	4	2.0%
p.Pro454Ser	0	2	2	1.0%
p.Leu307Phefs^*^5	5	0	5	2.5%
p.Asn493Ser	0	1	1	0.5%
p.Asn493Ser + T-107C	0	1	1	0.5%
c.293-13A/C>G + p.Val282Leu	1	1	2	1.0%
c.293-13A/C>G + p.Gln319Ter	0	1	1	0.5%
del 8 bp + p.Pro454Ser	0	1	1	0.5%
c.293-13A/C>G + p.Pro454Ser	0	3	3	1.5%
p.Pro454Ser + cluster ex6 + p.Ile172Asn	0	1	1	0.5%
prom.conv + p.Pro31Leu	0	2	2	1.0%
prom.conv + c.293-13A/C>G	0	2	2	1.0%
prom.conv + del 8 bp	0	1	1	0.5%
prom.conv + c.293-13A/C>G + p.Pro31Leu	0	1	1	0.5%
prom.conv + p.Pro31Leu + Als15Thr	0	2	2	1.0%
prom.conv + p.Leu307Phefs^*^5 + p.Gln319Ter	0	2	2	1.0%
p.Leu307Phefs^*^5 + p.Gln319Ter	1	0	1	0.5%
c.293-13A/C>G + cluster ex6 + p.Val282Leu + p.Gln319Ter	0	2	2	1.0%
Alleles together	80	120	200	

del, CYP21 gene deletion; prom conv, gene conversion in promoter region; conv, large or small; del 8 bp, deletion in exon 3; cluster ex6, cluster mutation in exon6.

In the study cohort, we found many alleles with multiple pathogenic variants: 6.5% of all alleles carried two pathogenic variants and 4% of alleles carried more than two pathogenic variants. The frequency of alleles with multiple pathogenic variants was higher in Slovenian patients (15.83% of all alleles). The most frequent pathogenic variant occurring on the same haplotype with another pathogenic variant was c.293-13A/C>G.

TARTs were identified in 7 of 24 male patients with available USG data, of whom all 7 (100%) patients had the SW form of CAH. (2 out of 7 Slovak patients and 5 out of 17 Slovenian patients with available USG data). The prevalence of TARTs was 29.2% in male patients with SW-CAH. All patients with TARTs had SW-CAH. Among patients with SV-CAH and NC-CAH, no TARTs were detected. The median age at diagnosis of CAH in patients with TARTs was 15 days. The median age at the diagnosis of TARTs was 13 years (range 8-24). Poor hormonal control due to non-compliance was present in all 7 patients. The average 17-OHP level at the diagnosis of TARTs was 217.88 nmol/l. **Table 3** shows the characteristics of patients with TARTs including their genotypes. As 2 boys were brothers, they have the same mutations.

**Table 3:** Characteristics of patients with TARTs.

Case/phenotype	*CYP21A2* haplotype (allele 1/allele 2)	Age at dg. of CAH	Age at dg. of TARTs	Tanner stage at dg. of TARTs	Hormonal control 17-OHP (ug/l)	Treatment non-compliance
Patient 1/SW-CAH	c.293-13A/C>G/c.293-13A/C>G	7 days	9 years	3	69.7 (poor)	yes
Patient 2/SW-CAH	c.293-13A/C>G/p.Leu307Phefs^*^5	26 days	8 years	2	70 (poor)	yes
Patient 3/SW-CAH	c.293-13A/C>G/c.293-13A/C>G + p.Pro454Ser	30 days	22	5	241.6 (poor)	yes
Patient 4/SW-CAH	c.293-13A/C>G/c.293-13A/C>G + p.Pro454Ser	7 days	24	5	330 (poor)	yes
Patient 5/SW-CAH	p.Pro454Ser + cluster ex6 + p.Ile172Asn/c.293-13A/C>G	15 days	10	5	660.5 (poor)	yes
Patient 6/SW-CAH	c.293-13A/C>G + p.Val282Leu/del	30 days	13	4	85.7 (poor)	yes
Patient 7/SW-CAH	c.293-13A/C>G + cluster ex6 + p.Val282Leu + p.Gln319Ter/prom.conv + p.Leu307Phefs^*^5 + p.Gln319Ter	9 days	19	5	67.70 (poor)	yes

TART, testicular adrenal rest tumour; CAH, congenital adrenal hyperplasia; SW-CAH, salt-wasting form; SV-CAH, simple virilizing form; NC-CAH, non-classic form; dg, diagnosis; del, CYP21 gene deletion; prom conv, gene conversion in promoter region; cluster ex6, cluster mutation in exon6; 17-OHP, 17-hydroxyprogesterone.

## 4 Discussion

In this study, we present a cohort of 100 individuals with CAH from Slovakia and Slovenia with genotype and phenotype information available. Since the population of the compared areas differs only by 400.000 inhabitants, we can say that we have compared two areas with similar demographic characteristics.

The distribution of the different forms of CAH was approximately the same for both countries. In both countries, SW-CAH was the predominant one in the cohort. However, the prevalence of SW-CAH was more frequent among Slovak patients than among Slovenian patients (72.5% versus 56.67%). NC-CAH was the second most frequent clinical form in both countries. The lower incidence of NC-CAH in the pediatric population may be explained by delayed diagnosis. When we compared the distribution of the different clinical forms of the disease in our cohort with the data from the other studies, we found differences in the distribution of SV-CAH and NC-CAH. In the compared sets, in 3 studies SV-CAH was the second most frequent and NC-CAH was the least frequent clinical form, while in the study by Marino et al. NC-CAH was more frequent than SV-CAH, similar to our data (3, 5–7).

The sex ratio for SW-CAH was balanced in the whole cohort. In NC-CAH the female sex was significantly predominant (85.71%), which can be explained by the fact that the manifestations of androgen excess are clinically clearer in women than in men, who can often be asymptomatic and diagnosed later in life (4).

In Slovak patients with SW-CAH (82.75% diagnosed after the introduction of NBS) the median age at diagnosis was 22.5 days lower than in the Slovenian patients. The difference was also large when comparing the median age at diagnosis of SW-CAH in boys from Slovakia and Slovenia (9 days versus 32.5 days). In Slovenian boys, the diagnosis of SW-CAH took four times longer than in females. This comparison confirms the importance of NBS, which has been in place in Slovakia since 2003, while it has not yet been introduced in Slovenia. During the analyses, we found that NBS in Slovakia most frequently detected patients with the SW form of the disease (92.59%), while the SV form was present in only 7.41% of the detected patients. In contrast, in patients not identified by NBS NC-CAH was predominant (46.15%).

In Slovak patients with SV-CAH, the median age at diagnosis was 0.91 years lower than in Slovenian group. This observation could be justified by earlier referral of patients from a general practitioner to a specialist or by parental notification of signs of gonadotropin-independent precocious puberty. Of course, a proportion of patients may also be detected by NBS. The age at diagnosis of NC-CAH was similar in Slovak and Slovenian patients.

The distribution of the most frequent pathogenic variants and the overall distribution of genotypes in the cohort was largely similar to the previously published European studies, whereas the differences were greater when compared to, for example, Mexican or Brazilian populations (8, 17). Deletion/large conversion and c.293-13A/C>G accounted for 55.5% of all pathogenic variants, followed by p.Ile172Asn and p.Val282Leu, both with a frequency of 8% (**Table 2**). The fifth most frequent pathogenic variant was p.Pro31Leu with a frequency of 4.5%. We found that c.293-13A/C>G was more frequent in Slovakia (37.5% versus 26.7%). The frequency of alleles with multiple pathogenic variants was higher in Slovenia (15.83% of all alleles), which may be the result of the founder effect. Clustering of point mutations on a single allele was previously reported in 1.9% of unrelated alleles in Dutch patients (6).

Direct sequencing of *CYP21A2* identified p.Asn493Ser substitution on both alleles and a concomitant T-307 pathogenic variant in the heterozygous form in a Slovenian patient with NC-CAH. The p.Asn493Ser substitution is also reported by some authors as a naturally occurring polymorphism, and by some as a disease-causing pathogenic variant, but its effect on the residual activity of the enzyme has never been analyzed *in vitro*. Ordonez-Sánchez et al. found a very high frequency of the p.Asn493Ser pathogenic variant in the Mexican population, and the proportion of homozygosity for the p.Asn493Ser substitution was higher in CAH patients than in the healthy population (17). Rodrigues et al. also reported a patient in whom the hemizygous Asn493Ser pathogenic variant was combined with the S268T pathogenic variant (18). It is possible that the Asn493Ser substitution may result in reduced enzymatic activity only when combined with the effect of another pathogenic variant.

A sporadic p.Arg357GIn pathogenic variant was found in a homozygous state in a Slovak patient with SV-CAH. This pathogenic variant with a residual enzymatic activity of 1.1% was first described in a Finnish study by Levo et al. (19) A Swedish study described 0.5% frequency of this pathogenic variant in the Swedish population studied, where it was associated with SW-CAH (20). In another Slovak patient, a rare pathogenic variant p.Arg409Cys was found in exon 10, previously found in two Brazilian patients, and *in vitro* testing resulted in an almost complete absence of enzymatic activity (21, 22). In a US study, the p.Arg409Cys pathogenic variant was associated with c.293-13A/C>G on the second allele and resulted in SV-CAH (22). In our cohort it led to SW-CAH with a *CYP21A2* deletion on the second allele. Other rare pathogenic variants included the p.Arg484Pro in exon 10, p.Arg317Ter in exon 8, which had not previously been described in the two populations studied. The relative frequency of the p.Arg484Pro pathogenic variant was very similar, 0.2% and 0.5% in the Chinese and US populations studied, respectively (23, 24). Based on a Taiwanese study, it can be assumed that the p.Arg317Ter pathogenic variant has a higher frequency in Taiwan in the Minnan population (25).

Many studies have investigated genotype-phenotype relationships in large national and multiethnic cohorts. In general, severe genotypes leading to SW-CAH showed a strong correlation with clinical phenotype. In our cohort, the overall genotype-phenotype correlation was high for severe pathogenic variants (0: 94.74%; A: 97.3%) but extremely low for group B genotypes (50%). A higher concordance was observed for group C genotypes (70.83%) compared to 2 European studies. In comparison (**Table 4**), previous studies reported 97-100% concordance for null, 79-96% for A, 46-87% for B, and 58-100% for C genotypes (3–6, 9). One-third of the patients (33.33%) with genotype B were classified as SW-CAH. Among the patients with genotype C, 25% of them had the classic form of CAH (SW and SV).

**Table 4:** Concordance of 0, A, B, C genotypes with assigned phenotypes compared to other studies.

Genotype/predicted phenotype	Our cohort (n=98)	USA (3) (n=1507)	Germany (2) (n=538)	Argentina (4) (n=454)	Netherlands (5) (n=198)	Poland (8) (n=44)
**0/SW-CAH %**	94.74	≤100	97	100	97	100
**A/SW-CAH%**	97.3	79	91	84	96	90.5
**B/SV-CAH %**	50	76	46	87	53	66.70%
**C/NC-CAH %**	70.83	>90	58	100	100	NA

CAH, congenital adrenal hyperplasia; SW-CAH, salt-wasting form; SV-CAH, simple virilizing form; NC-CAH, non-classic form.

The reasons for the observed differences are not fully elucidated. In some pathogenic variants, some residual enzyme activity may be present, for example in the case of c.293-13A/C>G, which led to SV-CAH (up to 20% of cases in our cohort), although it is classified as a severe pathogenic variant that usually leads to SW-CAH (3). Unusual chimeric genes are the cause of some genotype-phenotype discrepancies, and the sites of chimeric junctions resulting from genetic rearrangements may be of clinical significance. In addition to *CYP21A2* pathogenic variants, other genes that may affect the phenotype by modifying steroid action or salt balance include androgen receptor CAG repeat length; the highly polymorphic P450 oxidoreductase enzyme; splicing mutations in RNA splicing factors; and other genes encoding proteins other than the type II cytochrome P450 enzyme that have 21-OH activity (26). The role of *CYP2C19* as a potential modifier gene that contributes to extra-adrenal 21-hydroxylation of progesterone, which may alleviate mineralocorticoid deficiency in CAH, has been investigated. The results of a small Slovene study suggest that the CYP2C19*1/*17 genotype could lead to a very subtle modification of the clinical phenotype of 21-OH deficiency (27).

In addition, the effects of other pathways that regulate the severity of enzymatic loss, such as the posterior androgen pathway, which causes more heterogeneous phenotypes, may play a critical role in individual patients.

In genotype-phenotype correlation analysis, we observed increased clinical severity in compound heterozygotes who had one severe and one non-severe pathogenic variant, which would be expected to result in a milder phenotype (**Supplementary Table 2**). According to the study, compound heterozygotes with one classic pathogenic variant versus homozygotes with two mild pathogenic variants had a greater biochemical androgen response to ACTH stimulation. Various studies have reported varying levels of residual *in vitro* activity, which can range from 2-5% for p.Ile172Asn and 10-60% for p.Pro31Leu. This indicates ambiguity from a functional point of view (28–31). The finding of a p.Pro31Leu pathogenic variant associated with gene conversion in the promoter region could explain the increased severity of the phenotypes in Slovenian patients.

TARTs are a common complication in CAH male patients with a reported prevalence of 18.3-48% in the pediatric population, while the prevalence is higher in adult patients (32–35). In our cohort, the prevalence of TARTs was 29.2% in male patients with SW-CAH, which represented the most common form of CAH in our sample (63%). There was a slight difference in the prevalence of TARTs between the two countries: 2 patients out of 7 with available USG data in Slovakia and 5 out of 17 patients with available USG data in Slovenia. The overall prevalence of TARTs in our study (29.2%) was similar to the other studies (32–34).

Poor hormonal control was reported in 58% of studies examining CAH and TART (34). In our study all patients with TARTs were found to have poor metabolic control due to treatment non-compliance. In addition, studies found TARTs in patients with good metabolic control, too (36, 37). In the present study, the median age at the diagnosis of TARTs was 13 years, which was like the study of Aycan et al. and Kocova et al, where the median at the diagnosis of TARTs was 15.5 and 13.2 years respectively (33, 34). These 2 studies were describing the prevalence of TARTs in paediatric populations aged 2-18 years. In the study of Claahsen et al. most of the TARTs were detected in children above 10 years old (32). It may be suspected that hormones whose levels are elevated during puberty, such as luteinizing hormone (LH), additionally stimulate tumour growth. In one of the patients after improved compliance the ultrasound of the testes was without signs of TARTs. Other studies also proved the possible remission of TARTs if identified early if compliance and treatment regimen are improved (33, 34).

The relationship between the CAH genotype and the development of TART is unknown (38). Most of the published studies indicate that TARTs are associated only with classical forms of the disease, e.g. the null group carrying null mutations with no enzyme activity, A group containing c.293-13A/C>G variant with negligible enzyme activity, or group B composed of patients with a homozygous p.I172N mutation causing the SV form (31, 32, 34, 35). Since all patients with TARTs in our cohort had SW-CAH, they belonged to group null (4 patients) and group A (3 patients). The most frequent variant in patients with TARTs was c.293-13C>G, similar to the study by Aycan et al. (38) Given that genotype-phenotype correlation in CAH is not found in all patients (1), genetic analysis of larger TART patient cohorts may reveal additional genotypes associated with TART (35, 38).

One of the limitations of our study is, despite clear diagnostic criteria and careful data editing, we cannot exclude arbitrary overestimation of the severity of clinical phenotypes. Especially the cut-off limit of 4 years to diagnose NC-CAH in boys may affect the SV/NC-CAH ratio concerning phenotyping, since there are dilemmas with precise definition (3, 7, 14). It’s also important to mention that part of the patients was diagnosed more than 20 years ago, since then the definition of each form of CAH might have changed, too.

As our cohort includes a smaller set of especially Slovak patients, the frequency of less common mutations may not correspond to the full reality in the countries concerned. Regarding TARTs, we don’t have available all data that would allow the comparison of the screened and unscreened population (TART size, full hormonal profile, etc.), which would be beneficial to see in the future. Ideally, all patients should be evaluated by a single endocrinologist, which was not possible with patients originating from two countries.

In this study, we investigated the phenotype and genotype in the largest group of 21-hydroxylase-deficient CAH patients from Slovakia and Slovenia to date. In previous European studies, the number of Slovak patients with complete phenotype and genotype data did not exceed 20 and Slovenian 38, while our cohort included 40 Slovak and 60 Slovenian patients. In addition, our study compared two European countries, where in one NBS has been introduced in 2003, while in the other NBS is not used yet. Our study clearly demonstrated the importance of NBS which was particularly evident in the speed of diagnosis of severe forms of the disease in the critical neonatal period. This work may therefore support the introduction of NBS in Slovenia as well. In the future, it would be ideal to create a map of the prevalence of pathogenic variants, especially the less frequent ones, in the different regions of the countries studied. In this work, we did not include patients from the Central and Eastern Slovak regions, but due to the relatively significant population fluctuation towards the west, it may be assumed that the studied sample may represent the entire population of Slovakia.

Genetic diagnosis remains important in confirming or excluding the diagnosis in the case of unclear biochemical parameters, in segregation analysis - identifying the origin of the pathogenic variant, in determining carriers in siblings, and in prenatal diagnosis. Due to the observed high prevalence of TARTs in boys with CAH, annual screening by USG of testes for early detection and treatment is strongly recommended since there is possible remission of TARTs if identified early.

## Data availability statement

The original contributions presented in the study are included in the article/**Supplementary Material**. Further inquiries can be directed to the corresponding authors.

## Ethics statement

The studies involving human participants were reviewed and approved by National Medical Ethics Committee of Slovenia (No.: 0120-290/2021/3). Written informed consent to participate in this study was provided by the participants’ legal guardian/next of kin.

## Author contributions

RS: first authorship, data acquisition and interpretation, statistical analysis, drafting and revision of the manuscript. ZP: senior authorship, data interpretation, revision of the manuscript. UG: senior authorship, study design, data interpretation, revision of the manuscript. Other authors: revision of the manuscript. All authors contributed to the article and approved the submitted version.
