# Supplementary material for: Genetic and clinical characteristics including occurrence of testicular adrenal rest tumors in Slovak and Slovenian patients with congenital adrenal hyperplasia due to 21-hydroxylase deficiency
Source: Front Endocrinol (Lausanne). 2023 Mar 17;14:1134133. doi: 10.3389/fendo.2023.1134133 (PMC10064884; doi:10.3389/fendo.2023.1134133)
Supplement: Supplementary file 1 [file DataSheet_1.docx]

Supplementary Material

Genetic and clinical characteristics including occurrence of testicular adrenal rest tumors in Slovak and Slovenian patients with congenital adrenal hyperplasia due to 21-hydroxylase deficiency

Robert Saho^1^, Vita Dolzan^2^, Mojca Zerjav Tansek^4,5^, Andrea Pastorakova^6^, Robert Petrovic^6^, Maria Knapkova^7^, Katarina Trebusak Podkrajsek^2,8^, Jasna Suput Omladic^4,5^, Sara Bertok^5^, Magdalena Avbelj Stefanija^4,5^, Primoz Kotnik^4,5^, Tadej Battelino^4,5^, Zuzana Pribilincova^3^, Urh Groselj^4,5^

*** Correspondence:** Uhr Groselj [urh.groselj@kclj.si](mailto:urh.groselj@kclj.si)

Zuzana Pribilincova [zpribilincova@gmail.com](mailto:zpribilincova@gmail.com)

# Supplementary Figures and Tables

For more information on Supplementary Material and for details on the different file types accepted, please see [here](https://www.frontiersin.org/guidelines/author-guidelines#supplementary-material).

**Supplementary TABLE 1 |** *CYP21A2* haplotypes and clinical phenotypes of the cohort

| ***CYP21A2* haplotype (alelle 1 / alelle 2)** | **CAH phenotype** | | |
| --- | --- | --- | --- |
|  | **SW-CAH** | **SV-CAH** | **NC-CAH** |
| del / del | 8 | 0 | 0 |
| del / del 8 bp | 2 | 0 | 0 |
| del / p.Arg409Cys | 1 | 0 | 0 |
| del/conv / prom.conv | 1 | 0 | 0 |
| del / p.Leu307Phefs^*^5 + p.Gln319Ter | 1 | 0 | 0 |
| del / conv + c.293-13A/C>G + p.Pro31Leu | 1 | 0 | 0 |
| del / prom.conv + p.Pro31Leu + p.Als15Thr | 2 | 0 | 0 |
| p.Arg317Ter / c.293-13A/C>G + p.Val282Leu | 1 | 0 | 0 |
| p.Arg357Trp / c.293-13A/C>G | 1 | 0 | 0 |
| cluster ex6 / c.293-13A/C>G | 0 | 1 | 0 |
| p.Pro454Ser + cluster ex6 + p.Ile172Asn/ c.293-13A/C>G | 1 | 0 | 0 |
| c.293-13A/C>G / del/conv | 10 | 0 | 0 |
| c.293-13A/C>G + p.Pro31Leu / del/conv | 1 | 0 | 0 |
| c.293-13A/C>G / c.293-13A/C>G + p.Gln319Ter | 1 | 0 | 0 |
| c.293-13A/C>G / prom.conv + del 8 bp | 1 | 0 | 0 |
| c.293-13A/C>G + p.Val282Leu / del | 1 | 0 | 0 |
| c.293-13A/C>G / del 8 bp + p.Pro454Ser | 1 | 0 | 0 |
| c.293-13A/C>G + cluster ex6 + p.Val282Leu + p.Gln319Ter  / prom.konv + p.Leu307Phefs^*^5 + p.GIn318Ter | 2 | 0 | 0 |
| p.Leu307Phefs^*^5 / p.Leu307Phefs^*^5 | 1 | 0 | 0 |
| p.Leu307Phefs^*^5 / c.293-13A/C>G | 2 | 0 | 0 |
| p.Gln319Ter / c.293-13A/C>G | 2 | 0 | 0 |
| c.293-13A/C>G / p.Leu307Phefs^*^5 | 1 | 0 | 0 |
| c.293-13A/C>G / c.293-13A/C>G | 11 | 0 | 0 |
| c.293-13A/C>G / p.Arg355Cys | 1 | 0 | 0 |
| c.293-13A/C>G / c.293-13A/C>G + p.Pro454Ser | 2 | 0 | 0 |
| p.Ile172Asn / p.Ile172Asn | 0 | 1 | 0 |
| p.Ile172Asn/ del | 1 | 5 | 0 |
| p.Ile172Asn / c.293-13A/C>G | 3 | 2 | 1 |
| prom.konv / c.293-13A/C>G | 0 | 0 | 1 |
| p.Arg357GIn / p.Arg357GIn | 0 | 1 | 0 |
| p.Arg484Pro / c.293-13A/C>G | 1 | 0 | 0 |
| p.Pro31Leu / c.293-13A/C>G | 1 | 2 | 2 |
| p.Pro31Leu / p.Arg357Trp | 0 | 1 | 0 |
| p.Pro31Leu / p.Pro454Ser | 1 | 0 | 0 |
| p.Pro31Leu + prom.conv / del | 0 | 0 | 1 |
| p.Pro31Leu + prom.conv / + p.Gln319Ter | 0 | 1 | 0 |
| p.Val282Leu / c.293-13A/C>G | 0 | 1 | 2 |
| p.Val282Leu / p.Ile172Asn | 0 | 0 | 2 |
| p.Val282Leu / del/conv | 0 | 0 | 6 |
| p.Val282Leu / del 8 bp | 0 | 0 | 1 |
| p.Val282Leu / + p.Gln319Ter | 0 | 0 | 1 |
| p.Val282Leu / c.293-13A/C>G + p.Pro454Ser | 0 | 1 | 0 |
| p.Pro454Ser / del | 0 | 0 | 1 |
| p.Val282Leu / p.Pro31Leu | 0 | 0 | 2 |
| p.Asn493Ser / p.Asn493Ser + T-107C | 0 | 0 | 1 |
| **Together** | **63** | **16** | **21** |

Abbreviations: *CAH* congenital adrenal hyperplasia, *SW-CAH* salt-wasting form, *SV-CAH* simple virilizing form, *NC-CAH* non-classic form, *del* CYP21 gene deletion, *prom conv* gene conversion in promoter region, *conv* large or small, del 8 bp deletion in exon 3, *cluster ex6* cluster mutation in exon6

**Supplementary TABLE 2 |** Genotype-phenotype correlations in the cohort

| **Mutation group** | | | **Observed phenotype** | | | | | | **Relative frequency of the predicted phenotype** |
| --- | --- | --- | --- | --- | --- | --- | --- | --- | --- |
|  |  |  | **SW-CAH** | | **SV-CAH** | | **NC-CAH** | |  |
| **Allele 1** | **Allele 2** | **Predicted phenotype** | **SK** | **SI** | **SK** | **SI** | **SK** | **SI** |  |
| **0** | **0** | **SW-CAH (0)** | 7 | 11 | 0 | 1 | 0 | 0 | 94.74 % |
| **A** | **0** | **SW-CAH (A)** | 9 | 14 | 0 | 1 | 0 | 0 | 95.83% |
| **A** | **A** | **SW- CAH (A)** | 8 | 5 | 0 | 0 | 0 | 0 | 100.00% |
| **B** | **0** | **SV- CAH (B)** | 1 | 1 | 2 | 3 | 0 | 1 | 62.50% |
| **B** | **A** | **SV-CAH (B)** | 2 | 2 | 0 | 2 | 1 | 1 | 25.00% |
| **B** | **B** | **SV-CAH (B)** | 0 | 0 | 1 | 1 | 0 | 0 | 100.00% |
| **C** | **0** | **NC-CAH (C)** | 0 | 0 | 1 | 0 | 3 | 6 | 90.00% |
| **C** | **A** | **NC-CAH (C)** | 1 | 0 | 1 | 3 | 0 | 4 | 44.44% |
| **C** | **B** | **NC-CAG (C)** | 0 | 0 | 0 | 0 | 1 | 1 | 100.00% |
| **C** | **C** | **NC-CAH (C)** | 0 | 1 | 0 | 0 | 1 | 1 | 66.67% |
| **Unclassified (D)** | | | | | | | | |  |
| p.Arg355Cy s | **A** | **Unclassified (D)** | 1 | 0 | 0 | 0 | 0 | 0 |  |
| p.Asn493Ser | p.Asn493Ser + T-107C | **Unclassified (D)** | 0 | 0 | 0 | 0 | 0 | 1 |  |

Abbreviations: *CAH* congenital adrenal hyperplasia, *SW-CAH* salt-wasting form, *SV-CAH* simple virilizing form, *NC-CAH* non-classic form, *SK* Slovakia, *SI* Slovenia
